# Editorial: Historical-Cultural Psychology: The Contributions of Developmental Teaching in Different International Contexts

**DOI:** 10.3389/fpsyg.2021.810784

**Published:** 2021-12-09

**Authors:** Gustavo Cunha de Araújo, José Carlos Miguel, Yulia Solovieva

**Affiliations:** ^1^Department of Rural Education, Universidade Federal do Tocantins (UFT), Tocantinópolis, Brazil; ^2^Didactics Department, São Paulo State University (UNESP), Marília, Brazil; ^3^Faculty of Sciences for Human Development, Autonomous University of Tlaxcala, Tlaxcala, Mexico

**Keywords:** editorial, historical-cultural psychology, developmental teaching, Vygotsky (lev), Davydov

This Research Topic aimed to approach the area of developmental teaching from the perspective of Historical-Cultural Psychology, which could contribute to the global educational debate on the contribution of developmental teaching in different international contexts.

The articles published under this Research Topic range from theoretical and empirical studies on school-age children and theoretical reviews on studies conducted by Vygotsky and collaborators (such as Davydov) on young people and adults. The studies expand knowledge in this area and draw upon Historical-Cultural Psychology in analyses of and reflections on developmental teaching in different international contexts.

Vygotsky described this branch of psychology as being cultural (socially structured ways in which society organizes itself, with language as the instrument that facilitates the development of mental processes); instrumental (mediated nature of all complex psychological functions); historical (related to the cultural context, with the tools used by man in the environment emerging historically and improving over time, not emerging at once). Vygotsky emphasized the gap between science and the complex processes of the mind, outlining that it cannot be filled until we establish how natural processes and sensations relate to culture, which constructs the psychological functions of individuals.

Developmental teaching originated from the Historical-Cultural theory promoted by Vygotsky, focusing on the relationship between education and individual development. Although Vygotsky did not present the fundamentals of developmental teaching, David elaborated on this theory.

An important finding of Davydov's studies is that one of the main objectives of developmental teaching is to promote the development of thought and student autonomy through the performance of mental tasks and actions. In other words, Davydov states that when schools allow students to develop their thinking through the formation of scientific concepts, they contribute to their humanization, and consequently, to their full development.

We believe that developmental teaching is a way to promote the fuller development of school and university students. This can be achieved through a study activity that enables them to develop their higher psychological functions. This Research Topic is especially interesting and original owing to the participation of researchers from different institutions and countries with specific views “around” the cultural-historical approach and activity theory.

The first article by Erbil from Karamanoglu Mehmetbey University in Turkey, addresses the methods of cooperative learning and flipped classroom learning using Vygotsky's theory, and defends the use of inverted classrooms for education.

The theoretical review by Solovieva and Quintanar from the Autonomous University of Puebla and Tlaxcala in Mexico explores the gradual formation of actions in stages, outlining that they are one of the main contributions of the activity theory. The authors state that this methodology is fundamental for planning and organizing social and cultural interactions between children and adults, emphasizing the close relationship between their psychological development and the learning process. Conversely, they believe that better teaching methods based on the cultural-historical approaches and activity theory can be discovered.

The book review by Maidansky from Belgorod National Research University in Russia discusses notes on Vygotsky's studies analyzed by researchers Ekaterina Zavershneva and René van der Veer and published in that same book. According to Maidansky, these notes are still largely unknown in academic literature, as they deal with Jewish literature, for example. Maidansky also emphasizes that some studies in the book refer to a few internal conferences organized by Vygotsky to present his dissertations and the studies of collaborators.

The article by Sidneva from the Department of Psychology at Lomonosov Moscow State University in Russia analyzes the results of a study on Davydov's mathematical curriculum based on the assumptions of the Historical-Cultural Theory. The author found that children showed an improvement in their mathematical skills by participating in Davydov's mathematical curriculum program.

Furthermore, Clarindo et al. from Paulista State University (UNESP) in São Paulo, Brazil, investigated the use of learning activity as a means of developing children's theoretical thinking in the first grade. The authors found that learning activity can promote the development of higher psychic functions in school-age children, as it allows them to develop analysis, reflection, and mental planning skills.

The article by Veraksa et al., from the Faculty of Psychology at Lomonosov Moscow State University in Russia, investigates the relationships between certain aspects of classroom organization quality in groups of children aged 5–6 years. The authors found that children who interacted less in the classroom obtained high scores in cognitive flexibility tasks, while children who interacted more obtained high scores in memory and control tasks.

Finally, the article by de Araújo et al. from the Federal University of Tocantins and Paulista State University in Brazil, investigated how the consciousness of peasant young people and adults is formed by the visual and written elements of comic books. A didactic-formative experiment conducted with young people and adults found that the comic books produced by the participants allowed them to develop theoretical thinking, facilitating awareness of their reality, which is important in building a sense and understanding of meaning in the world around them.

The importance of the studies in this Research Topic lies in their contribution to facilitating the comprehension of aspects related to developmental teaching, activity theory, and higher psychic functions, among other relevant matters in Historical-Cultural Psychology. The studies provide a better understanding of human rationality and sociability based on an understanding of the interaction between an individual and their environment. Considering learning as a factor affecting development, these studies show that when the individual internalizes an object, it can be shared with other people in the form of language. However, this is only possible through the development of consciousness and the activity by which the individual has created material and immaterial instruments to communicate with the world.

This special edition allows us to understand the contribution of Historical-Cultural Psychology and Activity Theory to the topics of development and education, presenting theory, methodology, and research on a single platform.

## Author Contributions

GC, JC, and YS were responsible for designing the editorial, reviewing the analysis, and writing the manuscript. All authors contributed to the editorial and approved the submitted version.

## Conflict of Interest

The authors declare that the research was conducted in the absence of any commercial or financial relationships that could be construed as a potential conflict of interest.

## Publisher's Note

All claims expressed in this article are solely those of the authors and do not necessarily represent those of their affiliated organizations, or those of the publisher, the editors and the reviewers. Any product that may be evaluated in this article, or claim that may be made by its manufacturer, is not guaranteed or endorsed by the publisher.

